# 2465 Enhancing outcomes for young children with behavior disorders: A model for coordinated care

**DOI:** 10.1017/cts.2018.253

**Published:** 2018-11-21

**Authors:** Sufna G. John, Teresa Kramer, Nicola Edge, Michael Cucciare, Nicholas Long

**Affiliations:** 1 University of Arkansas Translational Research Institute; 2 Department of Psychiatry, University of Arkansas for Medical Sciences, Little Rock, AR, USA; 3 Department of Family and Preventative Medicine, University of Arkansas for Medical Sciences, Little Rock, AR, USA; 4 Department of Psychiatry, Division of Health Services Research, University of Arkansas for Medical Sciences, Little Rock, AR, USA

## Abstract

OBJECTIVES/SPECIFIC AIMS: (1) Identify current barriers to coordinated care between behavior consultation and PCIT services. (2) Identify current facilitators to coordinated care between behavior consultation and PCIT services. (3) Utilize this knowledge to create and pilot a coordinated care model that will enhance PCIT and behavior consultation service outcomes. METHODS/STUDY POPULATION: Objectives 1 and 2: Two focus groups consisting of 8–10 behavior consultants will be conducted to gather initial information on barriers and facilitators to coordinated care. Participants will be recruited from the state-funded behavior consultation team, to represent consultation occurring in rural and urban settings. All focus groups will be recorded and transcribed to capture questions and comments. Focus groups will be provided with an initial 10-minute overview of PCIT, including theory, prescribed strategies, and mode of intervention. A grand tour question will then be asked to elicit consultant perceptions of PCIT (e.g., “What are your thoughts on the compatibility between PCIT and behavior consultation services”), followed by probe questions deigned to elicit more detailed information about any perceived differences based on philosophical approach; differences in what is recommended in childcare settings Versus at home, etc.; and perceived barriers to coordinated care between school and outpatient services (e.g., “What factors make coordinating care with outpatient providers challenging?). Participants will be asked about their willingness to participate in a second focus group to review materials created to enhance coordinated care, based on their feedback. Objective 3. Based on feedback from the focus groups and quantitative data regarding factors associated with PCIT outcomes, we will develop an enhanced childcare component(s) for eventual implementation. To confirm our approach, we will invite the members of both focus groups back for a second session, in which we provide them with the created materials and elicit their feedback. We will start with a grand tour question (e.g., “How do you think parents and teachers would react to these materials?”) and then follow-up with probe questions related to feasibility (e.g., “How do you anticipate using these tools?”), appropriateness (e.g., “How adequately do you feel these materials address concerns with coordinated care?”), and acceptability (e.g., “How likely are you to begin using these tools within your consultation?”). Both focus groups will be recorded and transcribed to capture questions and comments. RESULTS/ANTICIPATED RESULTS: (1) Barriers and facilitators to coordinated care will include individual (e.g., acceptability of PCIT framework) and system-level factors (e.g., ease of communication between providers). (2) There will be significant overlap in coordination between the first phase of PCIT (which focuses on positive parenting strategies) and what is prescribed by behavior consultants. (3) There will be less compatibility between the second phase of PCIT (which focuses on disciplinary strategies) and what is prescribed by behavior consultants. (4) A coordinated are model will be rated as more feasible, appropriate, and acceptable to behavior consultants than PCIT services as currently prescribed. DISCUSSION/SIGNIFICANCE OF IMPACT: Childhood disruptive behaviors are among the most frequent reasons for referral to outpatient child/adolescent mental health clinics (Sukhodolsky *et al*., 2016). Disruptive and aggressive behaviors are problematic, not only for victims of children who are aggressive but also for aggressive children as they age. Although effective treatments exist, families are often provided with conflicting strategies for behavior management by outpatient clinicians and behavior consultants in the daycare setting, thus providing children inconsistent feedback which will delay their attainment of new skills. These data will provide the initial foundation for the development of a coordinated care model that promotes treatment efficacy by improving the compatibility between clinic-based PCIT and daycare-based behavior consultation services.

